# Genetic complexity of cassava brown streak disease: insights from qPCR-based viral titer analysis and genome-wide association studies

**DOI:** 10.3389/fpls.2024.1365132

**Published:** 2024-03-13

**Authors:** Leah Nandudu, Samar Sheat, Stephan Winter, Alex Ogbonna, Robert Kawuki, Jean-Luc Jannink

**Affiliations:** ^1^ School of Integrative Plant Sciences, Section of Plant Breeding and Genetics, Cornell University, Ithaca, NY, United States; ^2^ Root Crops Department, National Crops Resources Research Institute (NaCRRI), Kampala, Uganda; ^3^ Plant Virus Department, Leibniz Institute DSMZ-German Collection of Microorganisms and Cell Cultures, Braunschweig, Germany; ^4^ US Department of Agriculture, Agricultural Research Service (USDA-ARS), Ithaca, NY, United States

**Keywords:** cassava, food security, Ugandan cassava brown streak virus (UCBSV) titer, cassava brown streak virus (CBSV), genomics

## Abstract

Cassava, a vital global food source, faces a threat from Cassava Brown Streak Disease (CBSD). CBSD results from two viruses: Cassava brown streak virus (CBSV) and Ugandan cassava brown streak virus (UCBSV). These viruses frequently pose challenges to the traditional symptom-based 1-5 phenotyping method due to its limitations in terms of accuracy and objectivity. Quantitative polymerase chain reaction (qPCR) offers precise virus quantification, although high costs hinder its widespread adoption. In this research, we utilized qPCR to measure the viral titer/load of CBSV and UCBSV. The objectives were to evaluate titer variability within the Cycle 2 (C2) population in two different environments, establish connections between viral titers and CBSD severity scores from the 1-5 scoring method, perform Genome-Wide Association Studies (GWAS) to identify genomic regions associated with CBSV and UCBSV titers, and investigate the functional annotated genes. The results demonstrated a significantly higher prevalence of CBSV (50.2%) in clones compared to UCBSV (12.9%) with mixed infections in some cases. Genotypic effects, particularly concerning UCBSV, were significant, with genotype-by-environment effects primarily influencing CBSV titer. GWAS Studies identified genomic regions associated with CBSV and UCBSV titers. Twenty-one SNP markers on chromosomes 10, 13, 17, and 18 exhibited significant associations with CBSV titer, collectively explaining 43.14% of the phenotypic variation. Additionally, 25 SNP markers on chromosomes 1, 2, 4, 5, 8, 11, 12, 13, 16, and 18 were associated with UCBSV titer, and explained 70.71% of the phenotypic variation. No shared genomic regions were identified between CBSV and UCBSV viral titers. Gene ontology analysis also revealed diverse gene functions, especially in transport and catalytic activities. These findings enhance our understanding of virus prevalence, genetics, and molecular functions in cassava plants, offering valuable insights for targeted breeding strategies.

## Introduction

Cassava (*Manihot esculenta* Crantz) is a bushy shrub that originates from South America (Olsen and Schaal, 1999) and is a vital staple for millions globally. Its starchy storage roots provide essential carbohydrates and energy for people in Africa, Asia, and South America. Beyond food security, Cassava creates economic stability, particularly for small-scale farmers in sub-Saharan Africa and Asia as it is often cultivated for local markets. The crop also creates jobs and boosts rural economies (A Otekunrin and Sawicka, 2019), thus improving the quality of life for people along this economic chain. Besides rural economies, cassava’s derivatives meet global demands, driving commercial enterprises and income generation for farmers and entrepreneurs. Cassava starch finds applications in various industries like food processing, textiles, paper, pharmaceuticals, and biofuel production, enhancing its economic value (Li et al., 2017). Ultimately, cassava’s resilience in poor soils and drought conditions (Okogbenin et al., 2013; Orek et al., 2020) makes it a crucial resource for farmers combating the impacts of climate change. CBSD, ranked among the seven most serious threats to world food security (Pennisi, 2010; Patil et al., 2015) stands as a significant biotic challenge to cassava production, particularly in East, Central and Southern Africa, with the potential to cause losses of up to 100% in susceptible varieties (Hillocks et al., 2000; Hillocks and Jennings, 2003; Kaweesi et al., 2014). CBSD is caused by positive sense single-stranded RNA viruses belonging to the genus *Ipomovirus*, family *Potyviridae* (Winter et al., 2010; Walker et al., 2022). The two distinct virus species; cassava brown streak virus (CBSV) and Ugandan cassava brown streak virus (UCBSV), collectively referred to as cassava brown streak viruses (CBSVs), have unique genomes which contain, in addition to the typical potyvirus genes, a Ham1-like protein that is vital for the viruses to be able to infect cassava (Mbanzibwa et al., 2011). The viruses are transmitted by whiteflies (*Bemisa tabaci* (Genn.)) in a semi-persistent manner (Maruthi et al., 2017; Mero et al., 2021) while virus spread occurs mostly with the movement of infected stem cuttings by farmers which adds to the complexity of controlling the disease.

The most efficient and sustainable control of CBSD is genetic resistance in cassava. Screening for disease severity using a 1-5 visual score (Hillocks et al., 2000; Hillocks and Jennings, 2003; Yadav et al., 2011) is a well-established technique for cassava mosaic disease (CMD) resistance screening and amended for CBSD. It assigns scores based on the severity of symptoms on both leaves and storage roots, with scores ranging from 1 (indicating no visible symptoms) to 5 (indicating severe symptoms). Researchers have utilized this method to gain insights into how CBSVs manifest in diverse cassava varieties and under different environmental conditions thus guiding breeding programs whose aim is to develop robust and CBSD-resistant cassava varieties (Kaweesi et al., 2014; Kawuki et al., 2016; Masumba et al., 2017; Nzuki et al., 2017; Okul Valentor et al., 2018). However, despite progress made in identifying CBSD-tolerant and/or resistant varieties, this 1-5 scoring method has notable limitations. First, the symptom assessments are associated with subjectivity (Ozimati et al., 2021) thus affecting the consistency of scores among different evaluators. Second, the scores fail to identify cassava lines that do not show leaf or root symptoms but are infected with CBSVs (Munguti et al., 2021). Finally, CBSD symptom assessment cannot resolve the virus species, strains, or variants (Mohammed et al., 2012) which is vital information to understanding the unique responses of cassava to the distinct viruses and crucial for CBSD resistance breeding. Breeders generally assess aerial parts of plants, leaves, shoots and fruits and score for symptoms to define resistance. Absence of symptoms equals virus resistance, but this score can be given despite virus presence, replication, and systemic movement. For example, a most widely used resistance in tomato, ty1 confers resistance against Tomato Yellow Leaf Curl Virus (TYLCV), plants perform very well, show no symptoms on leaves, grow vigorously, and produce normal fruits while virus replication in ty1 resistant plants is maintained albeit at a lower level than in susceptible tomato. In contrast, CMD2 cassava, comprising the dominant CMD2 locus providing resistance to CMD, does not harbor mosaic virus infections, and asymptomatic plants do not maintain the virus (Chauhan et al., 2018).

Quantitative polymerase chain reaction (qPCR) has been used to quantify cassava brown streak virus titers in plants (Osman and Rowhani, 2006; Osman et al., 2007; Shirima et al., 2017; Luigi et al., 2023). It involves amplifying DNA, previously reverse transcribed from viral RNA (qRT-PCR), through repeated cycles of heating and cooling while a specific probe anneals to the PCR product generated emitting fluorescence signals corresponding to the amount of DNA amplified (TaqMan assay). The assay allows both a precise quantification of virus and because specific primers are used, a discrimination between CBSV and UCBSV. The method is the gold standard for detection and quantification of viruses at low amounts and in asymptomatic plants. In CBSD resistance breeding, qRT-PCR has been used to evaluate clones at advanced stages of breeding (Kaweesi et al., 2014; Shirima et al., 2017) and was an essential component of the workflow developed by Sheat et al. (2019) to identify CBSD resistance in South American cassava germplasm. However, qRT-PCR has not been widely used to complement CBSD phenotyping primarily because of its high costs and methodical requirements. Because the viruses associated with CBSD are not adequately assigned to a species and to a specific location, results from phenotyping at one location cannot be compared with those from another. Low virus titers in asymptomatic plants marking either an early stage of disease or resistance cannot be resolved, and this impacts the effectiveness of resistance screening. Therefore, integration of qRT-PCR to complement CBSD resistance screening is essential to improve precision and speed of breeding.

Using advanced molecular breeding tools, breeders and geneticists have investigated quantitative trait loci (QTL), conducted genome-wide association studies (GWAS) and identified candidate genes associated with CBSD phenotyping using symptom severity scores 1-5. GWAS facilitated the discovery of five single nucleotide polymorphisms (SNPs) located on cassava chromosomes 1, 13, and 18, revealing associations with CBSD foliar and root symptoms (Nandudu et al., 2023). Earlier, two genomic regions on chromosomes 4 and 11 were linked to CBSD foliar symptoms (Kayondo et al., 2018) with the GWAS hit on chromosome 11 associated with nucleotide-binding site leucine-rich repeat (NBS-LRR) genes, known for their crucial role in disease resistance. In another GWAS study, seven significant SNP markers on chromosome 11 were found to be related to mean root severity and disease index data (Kawuki et al., 2016). Other studies used biparental populations created by crossing Namikonga with Albert (Masumba et al., 2017), Kiroba and AR37-80 (Nzuki et al., 2017), NDL06/132 × AR37-80 (Kapinga, 2017) to identify QTLs associated with CBSD severity. One study identified nine QTLs on chromosomes 4, 5, 6, 11, 12, 15, 17, and 18, with distinct QTLs linked to CBSD foliar severity and root necrosis (Nzuki et al., 2017). Another study identified three QTLs on chromosomes 2, 11, and 18 associated with CBSD foliar and root severity. On chromosome 18, 27 annotated genes were identified, coding for Leucine Rich Repeat (LRR) proteins and signal recognition patterns (Masumba et al., 2017).

Ferguson et al., 2023 compared QTL from five bi-parental populations and identified QTL that were consistent across populations and identified candidate genes. The study by Ferguson et al., 2023 identified two QTLs for resistance to CBSD foliar symptoms on chromosome 4 and one on chromosome 11, along with an additional one on chromosome 18 for root necrosis. Among the candidate genes associated with these QTLs were Phenylalanine ammonia-lyase (PAL), Cinnamoyl-CoA reductase (CCR) genes, and three PEPR1-related kinases linked to the lignin pathway.

In this study, we aimed to identify potential genomic regions crucial for marker-assisted selection and genomic selection. We conducted a comprehensive analysis of CBSV and UCBSV titers. This approach streamlined the breeding process by enhancing the precision of phenotyping for marker-assisted breeding. The specific objectives of our study were to: (1) assess the variability in CBSV and UCBSV titers within a C2 population across two different environments, (2) establish phenotypic and genotypic correlations between CBSV and UCBSV titers and CBSD severity scores, (3) use GWAS analyses to identify genomic regions associated with CBSV and UCBSV titers and (4) provide insights into the functional annotated genes within the identified GWAS regions identified. Our study aimed to significantly advance cassava breeding strategies and enhance our comprehension of plant-virus interactions. This information serves as a guide for the development of markers essential for routine breeding.

## Materials and methods

### Plant material and field conditions

A cycle two (C2) population of genomic selection was developed at the National Crops Resources Research Institute (NaCRRI) Uganda. It incorporated two clonal evaluation trials (CETs) that were planted in two locations in 2019/2020 and 2020/2021. The C2 population resulted from successive cycles of selection and hybridization of clones selected based on genomic estimated breeding values (GEBVs) from cycle zero (C0) and cycle one (C1) populations (Ozimati et al., 2018; Ozimati et al., 2019). The C0 population comprised 52 clones obtained from the International Center for Tropical Agriculture (CIAT), the International Institute of Tropical Agriculture (IITA), and Tanzania’s national research program (Ozimati et al., 2018). Ninety-five (95) clones selected from C1 were crossed to create 6,570 seedlings. These seedlings were planted in an unreplicated trial in Namulonge and placed under natural virus infection by whiteflies with spreader rows of infected cassava TME204 as the source of inoculum for both CBSV and UCBSV. At harvest, 471 seedlings that had no visible CBSD symptoms provided planting material for subsequent CETs which were established at two high CBSD-pressure sites: Namulonge and Serere (Alicai et al., 2007; Kawuki et al., 2016; Ally et al., 2019). These trials were planted using an augmented incomplete block design, with each block containing three check varieties (UG110017, TME204, and Mkumba) known for their performance against CBSD. UG110017 and Mkumba are classified as tolerant, exhibiting less severe CBSD symptoms compared to the susceptible TME204. Each plot was made up of ten plants planted in a single row with 1 m spacing both within and between rows. Spreader rows of TME204 were also included to increase disease pressure across both environments.

### CBSD field evaluations

We used the 1-5 visual scoring scale (Legg and Thresh, 1998) for CBSD foliar and root symptoms to assess disease severity at 3, 6 and 12 months after planting (MAP). CBSD symptom severity assessments on leaves and stems were determined at 3 and 6 MAP, while root severity scores at 12MAP were based on the proportion of necrotic lesions in relation to the area of the cross-sectionally sliced root discs as described by Masumba et al., 2017. CBSD foliar incidence was recorded as a percentage based on the number of plants with symptoms divided by the total number of plants in a plot. Root necrosis incidence was obtained by dividing the number of storage roots that showed symptoms by the total number of storage roots in a plot.

### Virus titer quantification using qPCR

At harvest, root discs from each clone were collected, labeled, and dried overnight at 35°C. Subsequently, these dried discs were packaged in waterproof boxes and sent to the Plant Virus Department at the Leibniz Institute DSMZ-German Collection of Microorganisms and Cell Cultures in Braunschweig, Germany. Small pieces from dried cassava storage roots were collected in 2 ml Eppendorf tubes with stainless steel beads, placed in liquid nitrogen (N_2_), and stored at -80°C for later extraction. RNA from cassava storage roots was extracted using a kit (Epoch, USA). Samples were ground in a tissue lyser for tissue disruption (Qiagen TissueLyser LT, Germany), 450 µl of PRX buffer adjusted to 0.2% β-mercaptoethanol (Merck, Germany) was added to the powdered tissues and vortexed. Later steps were followed according to the manufacturer’s protocol. The integrity of the RNA was analyzed by gel electrophoresis in 2% agarose gels and RNA was quantified using a NanoDrop device (NanoDrop Spectrophotometer ND-1000, PEQLAB, Germany). Virus detection and quantification was conducted using a TaqMan assay (TaqMan Kit Maxima Probe/ROX qPCR Master Mix, Thermo-Fisher Scientific, USA) combining reverse transcription reaction and PCR in one step. COX (cytochrome oxidase) specific amplification was included as a plant control to test the performance of the reaction (Kaweesi et al., 2014). The qRT-PCR was done as described by Sheat et al., 2021 with primers used as described by (Adams et al., 2013; Kaweesi et al., 2014) as shown in **Table 1**. Reaction mixes for qRT-PCR contained 12.5 µl of Maxima Probe qPCR Master Mix (2x), 0.4 µM for COX primers and probe, 0.3 µM CBSV primers and probes or 0.4 µM UCBSV primers and probes, 5 µl of RNA preparation, 0.15 µl of Moloney Murine Leukemia Virus Reverse Transcriptase (M-MLV RT), and nuclease-free sterile water to a total reaction volume of 25 µl. Each RNA sample was analyzed in duplicate qRT-PCRs, and controls were included in every series. The One-step qRT-PCRs were incubated for 30 min at 43°C for complementary DNA (cDNA) synthesis, followed by an initial denaturation step for 2 min at 95°C and 40 cycles of denaturation (15 s at 95°C), annealing (30 s at 60°C), and synthesis (30 s at 72°C). qRT-PCR reactions were carried out in a qTOWER3 (Analytik Jena, Germany) equipped with qPCRsoft software to track the amplifications and check performance parameters. Cycle threshold (CT) values were used to calculate virus expression using the 2^-ΔΔCt method (Livak and Schmittgen, 2001) and COX as a reference gene for quantification. Virus expression was calculated relative to the virus titers measured in the infected susceptible TMS 96/0304.

**Table 1:** Sequences for CBSV, UCBSV and Cytochrome oxidase primers and probes.

Ugandan cassava brown streak virus	UCBSV forward	GATYAARAAGACITTCAAGCCTCCAAA	(Adams et al., 2013)
	UCBSV reverse	AATTACATCAGGRGTTAGRTTRT CCCTT	(Adams et al., 2013)
	UCBSV probe	FAM- TCAGCTTACATTTGGATTCCACGCT CTCA- TAMRA	(Adams et al., 2013)
Cassava brown streak virus	CBSV forward	GCCAACTARAACTCGAAGTCCATT	(Adams et al., 2013)
	CBSV reverse	TTCAGTTGTTTAAGCAGTTCG TTCA	(Adams et al., 2013)
	CBSV probe	FAM- AGTCAAGGAGGCTTCGTGC YCCTC -BHQ1	(Adams et al., 2013)
Cytochrome oxidase	COX forward	CGTCGCATTCCAGATTATCCA	(Kaweesi et al., 2014)
	COX reverse	CAACTACGGATATATAAGRRCCR RAACTG	(Kaweesi et al., 2014)
	COX probe	FAM-AGGGCATTCCATCCAGCGT AAGCA-TAMRA	(Kaweesi et al., 2014)

### DArTseq genotyping

Of the 471 clones in the CET, 320 were chosen at random at 12 MAP for genotyping and from each two young top leaves were collected from each seedling, folded, punched using a 5 mm hand puncher, and placed in 96-well plates. DNA extraction, genotyping and SNP calling were carried out for each sample using DArTseq genotyping platform (https://www.diversityarrays.com/technology-and-resources/dartreseq/). A total of SNP 28,434 markers were called, and these were combined with another imputed genotype dataset that consisted of common SNPs between DArTseq and GBS sequencing platforms (obtained from Marnin Wolfe, unpublished data), bringing the SNPs to 51,865. Combining both marker datasets improved SNP coverage. To increase the association power and account for the possibility of sequencing error, an additional filtering step was performed on the combined marker dataset to remove genotypes with >10% and SNPs with >5% missing data or with a minor allele frequency of less than 5%. A total of 30,846 SNP markers were obtained after filtering, and for downstream analyses, SNP markers were converted to the dosage format of 1, 0, -1, which represented alternative allele homozygotes, heterozygotes, and reference allele homozygotes, respectively.

### Statistical analyses

#### Broad-sense and narrow sense heritability

Two linear mixed effects models were fitted using *lme4* package in R (R Development Core Team 2016):


$${\text{y}}_{\text{ijc}}={\text{μ}}_{\text{i}:\text{c}}+{\text{g}}_{\text{i}:\text{c}}+{\text{β}}_{\text{j}}+{\text{r}}_{\text{i}:\text{c}\left(\text{j}\right)}+{\text{ε}}_{\text{ij}}\text{ Full model}$$

$${\text{y}}_{\text{ijc}}={\text{μ}}_{\text{i}:\text{c}}+{\text{g}}_{\text{i}:\text{c}}+{\text{β}}_{\text{j}}+{\text{ε}}_{\text{ij}}\text{ Reduced model}$$

Where y_ijc_ was a vector of phenotypic data, μ_i:c_ were fixed effects for the three checks and the population mean of the experimental clones with *i* indexing the checks and *c* indicating whether y_ijc_ is a check or an experimental clone. g_i:c_ are random effects of genotypes i with g_i_ ~ N (0, σ_g_^2^); β_j_ are random effects of year-location-incomplete block combination j with β_j_ ~ N (0, σ_β_^2^); r_i(j)_ are random effects of genotypes nested within year-location-incomplete block combination assumed to have a distribution of r_i:c(j)_ ~ N (0, σ_r_^2^_)_; and ε_ij_ is the residual with ε_ij_ ~ N (0, σ_e_^2^). Variances were partitioned, and broad sense heritability was calculated as H^2^ = σ _gi:c_^2^ / [σ _gi:c_^2^ + σ _ri:c(j)_^2^ + σ_εij_^2^]; where σ_gi:c_^2^ was the genotypic variance, σr_i:c(j)_^2^ variance of genotypes nested within the year-location-incomplete block combination and σ_εij_^2^ was model residual variance.

Narrow sense heritability was estimated using the function *emmreml* in the *EMMREML* package (Akdemir and Okeke, 2015) in R. Narrow sense heritability was calculated using h^2^ = σ _Zi:c_^2^/ [σ _Zi:c_^2^ + σ_εij_^2^]; where σ _Zi:c_^2^ was additive variance and σ_εij_^2^ was the model residual variance.

#### Trait correlations

Trait correlations of CBSV titer, UCBSV titer, CBSD incidence, and severity traits at 3, 6, and 12 MAP (CBSDi3, CBSDi6, CBSDi12, CBSDs3, CBSDs6, and CBSDs12) were evaluated based on phenotypic values and BLUPs. All analyses were performed using the *cor* function in R package (R Development Core Team 2016), and visualization of the correlation matrices was done using the ‘*corrplot*’ R package (Wei and Simko, 2017).

#### Genome wide association studies

GWAS was carried out using GAPIT (Lipka et al., 2012). A mixed linear model from GAPIT was fitted to analyze viral titers of CBSV and UCBSV while accounting for population structure using five principal components (PC), kinship relationships via the genomic relationship matrix (K), and environmental variables (year and location effects), treated as covariates. The Bonferroni correction (0.05/total number of markers) was used to identify significant SNPs. Percentage of variation explained by the significant SNPs was calculated as a multi-kernel model in the *EMMREML* package (Akdemir and Okeke, 2015) in R.

#### Candidate gene analysis

Significant SNP markers linked to either CBSV or UCBSV viral titer were used to determine genomic regions that were characterized for candidate genes. Gene positions were established using *M. esculenta* genome version 6 (Bredeson et al., 2016), and any genes that overlapped with these significant genomic regions were classified as candidate genes. BEDTools were employed to detect potential genes in a window of 1 Mb up- and down-stream of the identified significant SNPs in GWAS findings (Quinlan and Hall, 2010). Identified genes were characterized for gene ontology including molecular and biological functioning using PANTHER version 17.0 (Mi et al., 2019) and M. esculenta genome version 6 gene ontology database in Phytozome (Goodstein et al., 2012).

## Results

### Phenotypic variation for CBSV and UCBSV virus titer

In the combined dataset, CBSV was more frequently detected compared to UCBSV. Specifically, CBSV was detected in 50.2% of clones in the C2 population while UCBSV was detected in 12.9% of the clones. Notably, the occurrence of UCBSV was lower in Serere, with a detection rate of 1.5% in the 2019 season and 14% in the 2020 season. Conversely, at the Namulonge experimental site, the UCBSV detection rates were 10.2% and 23.8% for the respective years (**Table 2**). Mixed infections were observed in 77 clones in both Namulonge and Serere, amounting to a total of 8.16% across the two years. Namulonge exhibited higher prevalence rates of 6.01% in the 2019/2020 season and 17.25% in the 2020/2021 season, whereas Serere had rates of 1.01% and 6.76% for the respective seasons.

**Table 2:** Presence and absence of CBSV and UCBSV in the C2 population across two growing seasons.

Location	Year	CBSV (%)		UCBSV (%)		Mixed CBSV and UCBSV (%)	
		(+)	(+)	(+)	(-)	(+)	(-)
Namulonge	2019	43.61	10.19	10.19	89.81	6.01	93.99
	2020	61.72	23.83	23.83	76.17	17.25	82.75
Serere	2019	39.70	1.51	1.51	98.49	1.01	98.99
	2020	54.26	13.90	13.90	86.10	6.76	93.24
Combined data	2019/2020	50.21	12.94	12.94	87.06	8.16	91.84

(+) = presence of C/UCBSV; (-) = Absence of C/UCBSV; CBSV titer = Cassava brown streak virus titer and UCBSV titer = Uganda cassava brown streak virus titer.

### Partitioning of phenotypic variance explained by genotype, environment, and genotype-by-environment interactions

The full model exhibited significantly lower deviance values (P ≤ 0.001) compared to the reduced model in relation to both CBSV and UCBSV titers (**Table 3**). Differences were also observed in how genotype, environment, and genotype-by-environment (G x E) interactions contributed to overall phenotypic variance. Specifically, the genotype contributed 1.4% and 92.4% of the observed phenotypic variance while the genotype-by-environment effects contributed 92.7% and 7.2% for CBSV and UCBSV titers, respectively (**Table 4**). Environmental factors contributed less than 2% of the total phenotypic variance for both CBSV and UCBSV titers. Broad-sense heritability estimates of 0.03 (CBSV) and 0.96 (UCBSV) were observed for virus titers, highlighting that UCBSV titer is primarily influenced by genetics.

**Table 3:** A chi-square test comparing the deviance values for G x E model (Full Model) with a model fitted without G x E term (Reduced model).

Deviance values for CBSV and UCBSV viral titer†			
Traits	Full-GxE model	Reduced-GxE model	Chi-sq Test
CBSV titer	10388	12223	1834.6***
UCBSV titer	9213	9925	712.01***

***, significant at probability level of 0.001; and †Deviance and chi-square values for CBSV titer = Cassava brown streak virus titer and UCBSV titer = Uganda cassava brown streak virus.

**Table 4:** Allocation of variance components and broad-sense heritability estimates derived from the full model.

Proportion of variance (%) explained by Genotype, Environment and G*E				Broad-sense heritability
Trait	Genotypic effects	Environment effects	G*E effects	H^2^
CBSV titer	1.43	1.27	92.68	0.03
UCBSV titer	92.42	0.26	7.20	0.96

CBSV titer = Cassava brown streak virus titer and UCBSV titer = Uganda cassava brown streak virus titer.

### Correlation between CBSV and UCBSV titer with CBSD severity scores from the 1-5 scoring method

**Figure 1:**
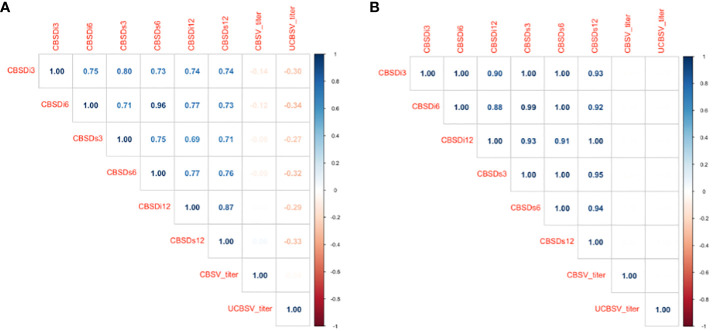
**(A)** Pearson’s phenotypic correlations between CBSV titer, UCBSV titer, incidence, and severity scores from the 1-5 scoring method. **(B)** Pearson’s genotypic correlations between CBSV titer, UCBSV titer, incidence, and severity scores from the 1-5 scoring method. CBSV titer = Cassava brown streak virus titer and UCBSV titer = Uganda cassava brown streak virus titer, CBSDi3 = cassava brown streak foliar incidence at 3 months after planting (MAP); CBSDi6 = cassava brown streak foliar incidence at 6 MAPS; CBSDs3 = cassava brown streak foliar severity at 3 MAP; CBSDs6 = cassava brown streak foliar severity at 3 MAP; CBSDi12 = cassava brown streak root incidence; CBSDs12= cassava brown streak root severity.

The magnitude of phenotypic and genotypic correlations between CBSV and UCBSV titer did not vary (**Figures 1A, B**). CBSV and UCBSV titers were not correlated, and this was consistent across the two years in the Namulonge and Serere experimental sites. Likewise, the correlations involving CBSV titer, UCBSV titer, CBSD incidence, and severity scores from the 1-5 scoring scale ranged from 0 to -0.34 for phenotypic correlations, while the genotypic correlations remained consistently at zero. Phenotypic, and genetic correlations between CBSD incidence and severity scores were positive ranging 0.69 to 1 (p< 0.001).

### Genome-wide association studies

**Figure 2:**
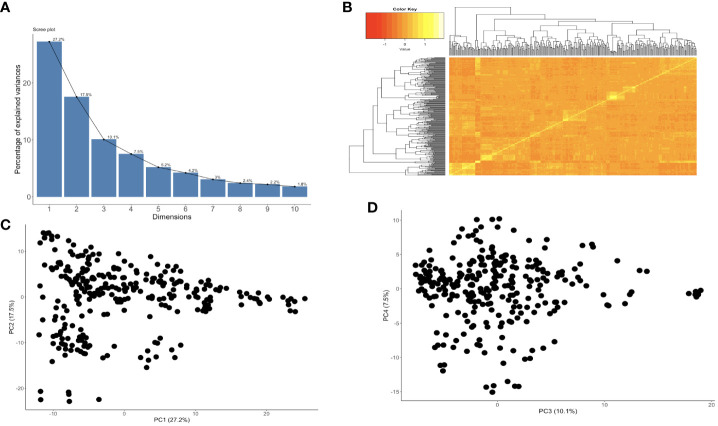
**(A)** The proportion of genetic variation explained by the first 10 principal components from 30,846 SNP markers and 302 cassava clones that were in two years and two locations, **(B)** kinship plot showing the relationship among the genotypes., **(C)** Variance explained by PC1 and PC2, and **(D)** Variance explained by PC3 and PC4.

**Figure 3:**
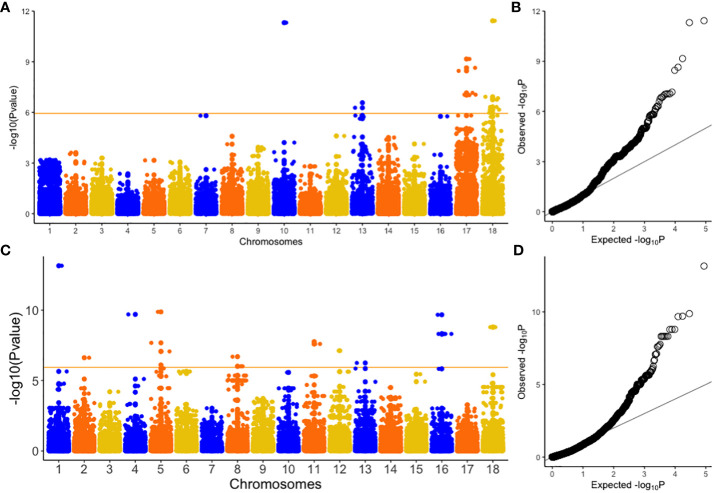
Manhattan and Quantile-quantile (Q-Q) plots of genome-wide association studies for CBSV **(A, B)** and UCBSV **(C, D)**. CBSV titer = Cassava brown streak virus titer and UCBSV titer = Uganda cassava brown streak virus titer. Orange horizontal line indicates Bonferroni genome wide significance level [-log_10_(0.05/number of markers)].

GWAS was performed on 302 cassava genotypes and 30,846 SNP markers using a mixed linear model (MLM) with GAPIT. Genomic background effects were also modeled via a marker inferred Kinship matrix and were displayed as a heatmap where yellow indicated the highest correlation between a pair of individuals and the red the lowest correlation (**Figure 2B**). Population structure was accounted for using five (5) principal components that all explained 67.6% of the total phenotypic variance (**Figures 2A, C, D**). A Bonferroni correction threshold (0.05/total number of markers (n)) was used to discover significant genomic regions that were associated with both CBSV and UCBSV viral titer (**Figure 3**). A total of 21 SNP markers on chromosomes 10, 13, 17, and 18 showed significant associations with CBSV titer, collectively explaining 43.1% of the phenotypic variation (**Table 5**). The individual SNP markers explained proportions of variance ranging from 1.8% to 3.8%. Moreover, 25 SNP markers located on chromosomes 1, 2, 4, 5, 8, 11, 12, 13, 16, and 18 were associated with UCBSV titer, collectively explaining 70.7% of the phenotypic variation. These specific SNP markers inferred variances ranging from 15.3% to 72.9%, surpassing the phenotypic variance explained by significant SNP markers associated with CBSV titer. There were no shared genomic regions identified between CBSV and UCBSV viral titer. GWAS was also performed on severity scores obtained from the 1-5 scoring method, using CBSV and UCBSV titer as covariates. No significant genomic regions were identified.

**Table 5:** Single nucleotide polymorphism (SNPs) locations associated with CBSV and UCBSV viral titer in the C2 population.

Trait	SNP	Chromosome	Position	-log10 (p-value)	Proportion of variance explained by significant SNP (%)	R^2^
CBSV titer	S10_28577329	10	28577329	11.3172242	2.0	0.2
	S13_12581595	13	12581595	6.57125932	2.3	0.1
	S13_14509619	13	14509619	6.27303911	2.6	0.1
	S17_19901143	17	19901143	7.04954338	1.8	0.1
	S17_20082509	17	20082509	9.16366732	2.6	0.2
	S17_20438858	17	20438858	7.04954338	1.8	0.1
	S17_20470971	17	20470971	7.04954338	1.8	0.1
	S17_20598926	17	20598926	7.020699	2.0	0.1
	S17_20939033	17	20939033	8.64101604	2.3	0.2
	S17_23685241	17	23685241	7.15483604	1.9	0.1
	S17_24274578	17	24274578	8.45892322	2.5	0.2
	S18_10256940	18	10256940	6.9241827	3.3	0.1
	S18_10987917	18	10987917	5.98007266	2.2	0.1
	S18_12726378	18	12726378	6.15729631	2.3	0.1
	S18_12726819	18	12726819	11.4309126	3.8	0.2
	S18_12758821	18	12758821	6.7484451	2.0	0.1
	S18_12765548	18	12765548	6.15729631	2.3	0.1
	S18_12881902	18	12881902	6.83982091	2.0	0.1
	S18_13269088	18	13269088	6.83982091	2.0	0.1
	S18_13447229	18	13447229	6.31843511	1.9	0.1
	S18_13905879	18	13905879	6.31843511	1.9	0.1
UCBSV titer	S1_4442142	1	4442142	13.1554814	–	1
	S2_13591693	2	13591693	6.61862394	28.9	0.4
	S4_755752	4	755752	9.6986525	58.1	0.7
	S5_1572605	5	1572605	9.87635619	47.0	0.7
	S5_1961417	5	1961417	7.67523093	52.1	0.5
	S5_5740165	5	5740165	7.06721541	26.5	0.5
	S5_5744580	5	5744580	7.06721541	26.5	0.5
	S5_5755199	5	5755199	6.10510206	20.7	0.4
	S8_919385	8	919385	6.00723876	15.3	0.4
	S8_919402	8	919402	6.00723876	15.3	0.4
	S8_939712	8	939712	6.69403613	19.1	0.4
	S11_22839054	11	22839054	7.59760624	43.7	0.5
	S11_22855976	11	22855976	7.59760624	43.7	0.5
	S11_30287604	11	30287604	7.7611451	52.1	0.5
	S12_1300480	12	1300480	7.11990669	31.1	0.5
	S13_27124783	13	27124783	6.24722706	31.1	0.4
	S16_29660229	16	29660229	9.67199322	45.0	0.7
	S16_26074428	16	26074428	8.31134573	72.9	0.6
	S16_26074452	16	26074452	8.31134573	72.9	0.6
	S16_26074516	16	26074516	8.31134573	72.9	0.6
	S16_26074535	16	26074535	8.31134573	72.9	0.6
	S16_26074556	16	26074556	8.31134573	72.9	0.6
	S16_26074558	16	26074558	8.31134573	72.9	0.6
	S18_8478449	18	8478449	8.79094556	49.8	0.6
	S18_8496771	18	8496771	8.79094556	49.8	0.6

### Candidate gene analysis

A total of 207 genes were discovered within the significant genomic regions associated with both CBSV and UCBSV titer (**Supplementary Table 1**). These genes were categorized using *M. esculenta* annotation IDs from version 6 of the cassava genome, focusing on molecular function and cellular components. In terms of molecular functions, several of the genes (36.6%) remained uncharacterized. The characterized genes were grouped into twelve categories: (1) 5.4% transporter activity (GO:0005215), (2) 0.6% translation regulator activity (GO:0045182), (3) 5.9% transcription regulator activity (GO:0140110), (4) 33.3% catalytic activity (GO:0003824), (5) 3% molecular function regulator activity (GO:0098772), (6) 0.4% cytoskeletal motor activity (GO:0003774), (7) 3.1% ATP-dependent activity (GO:0140657), (8) 2.3% molecular transducer activity (GO:0060089), (9) 0.4% molecular adaptor activity (GO:0060090), (10) 0.5% antioxidant activity (GO:0016209), (11) 2.4% structural molecule activity (GO:0005198) and (12) 25.4% binding (GO:0005488) (**Supplementary Table 2**). Regarding cellular components, most genes were uncharacterized. However, two distinct categories stood out: cellular anatomical entity (62%) and protein-containing complex (14%) (**Supplementary Table 3**). It’s worth noting that no significantly overrepresented Gene Ontology (GO) terms were found, likely due to the diverse molecular processes covered by the potential candidate genes in this study, resulting in a heterogeneous set of genes.

## Discussion

CBSD severely impacts cassava production in East and Central Africa. Research to identify immune or resistant varieties began in the 1930s in Tanganyika (modern-day Tanzania) using the 1-5 symptom-based scoring method (Tomlinson et al., 2018). Tolerant varieties like Namikonga, Kiroba, NASE 1, NASE 14, and NASE 19 initially remained symptom-free but eventually succumbed to CBSD due to the repeated use of infected stem cuttings, leading to increased virus accumulation (Kaweesi et al., 2014; Kawuki et al., 2016). However, the persistent vulnerability of elite clones to CBSD is changing with the discovery of CBSV-resistant clones in South American germplasm, identified through a q-RTPCR screening strategy (Sheat et al., 2019). Some of these clones exhibit a differential resistance response to CBSD, with resistance to CBSV but susceptibility to UCBSV noted in certain cases (Sheat and Winter, 2023). These varying responses add complexity to distinguishing between CBSV and UCBSV using phenotypic data from the 1-5 visual scoring scale. qRT-PCR has been expanded as a screening tool to assess breeding populations resulting from crosses with CBSV-resistant clones from South America, currently under evaluation in the NextGen project in Germany and Africa (Sheat et al., 2022; Sheat and Winter, 2023). Data from these evaluations will be pivotal in the ongoing efforts to enhance CBSD resistance. In other studies, viral titer has proven valuable for correlating symptom expression, particularly in tolerant clones like Namikonga and susceptible ones like Albert (Sheat et al., 2019). It has also been utilized to characterize the mechanism of action in highly resistant South American clones, specifically DSC 167 (Col 2182) (Sheat et al., 2019; Sheat et al., 2022). Despite these insightful applications, q-RTPCR has not yet been integrated into routine CBSD resistance breeding programs in Africa. This underscores the importance of adopting this sensitive method and emphasizing viral titer/load as an additional tool for phenotyping in the development of resistant cassava varieties, with a specific focus on the infection process. Limited knowledge exists about the genomic regions and candidate genes associated with CBSV and UCBSV viral titers, particularly in their interaction with severity scores using the 1-5 method. Understanding these genetic aspects is critical for precise CBSD control strategies, including marker-assisted selection and genomic selection, to enhance genetic progress. Phenotyping CBSD through viral titer measurement will aid in selecting low or virus-free cassava clones, reducing inoculum sources, and limiting CBSD spread. While most qPCR studies focused on a limited number of clones (Kaweesi et al., 2014; Shirima et al., 2017; Sheat et al., 2019; Shirima et al., 2019; Sheat et al., 2021), our research employed qRT-PCR to assess CBSV and UCBSV viral titer in 471 clones within the C2 population over two years and at two locations. By conducting GWAS and candidate gene analysis, our goal was to dissect the genetics of cassava brown streak disease pathogenesis. These findings will be integrated into Uganda’s cassava breeding program and other regional initiatives to formulate targeted CBSD control strategies, including the selection and deployment of CBSD species-specific immune/resistant/tolerant clones.

Results from our study revealed that CBSV was more widespread than UCBSV (Mbanzibwa et al., 2011), and both viruses displayed a consistent rise over the two evaluation years. This continuous rise in CBSV and UCBSV incidence can be linked to the reuse of planting materials across growing seasons. This means that if an infected genotype’s cutting from the first year was used in the second year as planting material, then the CBSD infection would be systemic and would be maintained in the second year. The infected planting material from the first year also serves as a source of inoculum for new infections along with the spreader rows in the second season, thus increasing the incidence of CBSD. Reusing planting materials from the 2019/2020 growing season to the 2020/2021 season could be responsible for the differences between growing seasons and how various genotypes responded to CBSV species (Shirima et al., 2019). Other studies have also shown that UCBSV faces replication restrictions within the host (Amuge et al., 2017; Sheat et al., 2021), which is a recognized resistance mechanism, unlike CBSV. Consequently, CBSV titer, owing to its unrestricted replication, is more readily available, and whiteflies can easily access its inoculum (Maruthi et al., 2017). This ease of access facilitates its transmission and higher incidence when compared to UCBSV. Specifically, the results of our study showed that Namulonge had significantly higher incidences of CBSV (43% and 62%) and UCBSV (10% and 24%) during the 2019 and 2020 growing seasons, respectively, compared to Serere. This observation is consistent with prior findings based on viral titer assessments (Kaweesi et al., 2014) and CBSD symptom evaluation using the visual 1-5 scoring method (Okul Valentor et al., 2018; Nandudu et al., 2023), and reaffirms Namulonge as a significant CBSD hotspot. Namulonge has also consistently shown high whitefly densities (Gwandu et al., 2019), which are known vectors of CBSVs.

In the C2 population, CBSV and UCBSV infections led to significant phenotypic variability influenced by genetics, the environment, and genotype-environment interactions. UCBSV titer was mainly influenced by genetics (92.5%), indicating substantial variation within the C2 population. This observed UCBSV titer variability suggests the suitability of the C2 clone pool for genetic analyses. In contrast, CBSV titer showed stronger genotype-environment interactions, indicating expression variability of genotypes in Namulonge and Serere. The significant impact of genotype-by-environment effects on CBSV viral titer suggests the need for multi-location trials to identify stable genotypes for genetic studies with clones in the C2 population. The high incidences of CBSV titer observed in this study can be attributed to the intricate genotype-by-environment interactions, indicating that specific environmental factors significantly influence the increased CBSV occurrence. These genotype-by-environment interactions could arise from whitefly populations that feed on infected plants that are highly likely to be infected from CBSV compared to UCBSV. This preference could also elucidate the increased incidence of CBSV in Namulonge and Serere over a span of two years (Maruthi et al., 2017; Gwandu et al., 2019; Shirima et al., 2019). Environmental effects had the smallest impact on both CBSV and UCBSV titer phenotypes, and this was consistent with previous studies on CBSD severity scores in the C2 population (Nandudu et al., 2023), farmer-preferred cassava clones in Uganda (Tumuhimbise et al., 2014; Anthony et al., 2015) and sixty-four landraces in Tanzania (Masinde et al., 2018). Also, a significantly low broad-sense heritability estimate of 0.03 for CBSV titer which contrasted with the high 0.96 estimate for UCBSV titer, underscores the predominant genetic influence on observed UCBSV titer variations. This novel UCBSV heritability finding aligns with the established 1-5 scoring method and correspond with heritability estimates for CBSD severity traits in previous research (Kayondo et al., 2018; Okul Valentor et al., 2018; Nandudu et al., 2023; A. Ozimati et al., 2019).

Moderate to high heritability estimates of 15% to 96% have been noted for CBSD severity traits (Nandudu et al., 2023). Similarly, moderate to high estimates have been reported in two breeding panels, some of which constituted the C0 population (Kayondo et al., 2018).

Phenotypic and genetic correlations were calculated for CBSV and UCBSV titer, CBSD incidence scores, and severity scores obtained through the 1-5 scoring method. Results revealed a range of phenotypic correlations from 0 to -0.34, while the genotypic correlations consistently remained at zero. These findings suggest the presence of separate resistance mechanisms governing both CBSD foliar and root symptoms and virus accumulation and highlight that some clones could be asymptomatic but can still support virus replication. This finding contrasts with the results reported by (Kaweesi et al., 2014), where genotypes showing CBSD foliar symptoms exhibited strong correlations with either CBSV or UCBSV viral titer. However, in the case of root necrosis severity, correlations varied across clones, with many showing no significant correlations (Kaweesi et al., 2014). Although there were no direct correlations between CBSD foliar and root symptoms and viral titer/load in our study, other studies have observed correlations between CBSV titer in foliar and storage root tissues (Moreno et al., 2011). High CBSV titer in leaves suggested a potential link to elevated CBSV titer in storage roots (Moreno et al., 2011). However, clones that restricted CBSD viruses in the root were also identified exhibiting symptoms and harboring elevated virus concentrations in tuberous storage roots, while the shoots remained free from the virus (Sheat et al., 2019; Sheat et al., 2021). For this reason, plant breeding programs when evaluating their germplasm for CBSD resistant clones, should consider virus titer accumulation in both leaves and storage roots during the screening process (Kaweesi et al., 2014).

We used GWAS to analyze the genetic basis of CBSV and UCBSV titers in the C2 population for genomic selection. A total of 46 significant SNPs were associated with CBSV and UCBSV titer, with no shared SNP markers between both traits. The significant SNPs linked to the CBSV titer explained 43.1% of the variance, with individual SNP markers contributing between 1.8% and 3.8% variance. This indicates that the SNPs associated with CBSV were not derived from a major gene but rather from multiple genes exerting minor effects on the phenotype. SNPs linked to UCBSV titer collectively accounted for 70.7% of the phenotypic variation. Individual SNP markers contributed between 15.3% and 72.9% variance, with SNPs on chromosome 16 explaining the highest proportion. This implies that specific SNPs, especially those accounting for substantial variations in the phenotype, might be linked to several major genes that have moderate to significant effects on the phenotype. Contrastingly, SNPs responsible for smaller variations in the UCBSV phenotype might be linked to multiple genes exerting minor effects on the phenotype. Therefore, much like the severity scores from the 1-5 previously identified as quantitative traits (Kayondo et al., 2018), both CBSV and UCBSV titers exhibit the defining characteristics of quantitative traits. This means that their variations are continuous and influenced by multiple genetic factors rather than being determined by a single gene. Our study’s findings concerning the genomic regions associated with both CBSV and UCBSV titers are consistent with previous GWAS and QTL analyses that focused on the severity of CBSD using the 1-5 scoring method. These shared genomic regions have been identified in various studies, including chromosomes 4 and 11 (Ferguson et al., 2023), chromosome 1 (Nandudu et al., 2023), chromosomes 4 and 11 (Kayondo et al., 2018), chromosomes 4, 5, 11, 12, 17, and 18 (Nzuki et al., 2017), chromosomes 2,11 and 18 (Masumba et al., 2017), and chromosome 11 (Kawuki et al., 2016). This observation suggests that specific gene(s) closely located on these chromosomes could potentially have an influence on CBSV and UCBSV titers, as well as the severity of CBSD. It is worth noting that these genes might have originated from important founder varieties, such as Namikonga and Kiroba, which have been extensively utilized in CBSD resistance breeding within numerous cassava breeding programs. These consistent findings across different studies reinforce the significance of these genomic regions in the context of CBSD and highlight their potential importance for further research and breeding programs.

This study examined molecular functions and cellular components of genes within the identified GWAS regions of interest. These functions include facilitating the movement of substances within or between cells, regulating gene transcription, catalyzing biochemical reactions, generating force for movement, binding molecules, and maintaining structural integrity within cellular complexes.

These diverse functions underscore the complexity of the identified GO and highlight their significance at the molecular and cellular levels during the initiation, establishment, and progression of viral infections. One GO of interest was cytoskeletal motor activity (GO:0003774), a function recognized for generating force essential for various movements (Stewart et al., 2014; Houdusse, 2020), including muscle contraction in whiteflies’ adaptation to tobacco (Xia et al., 2017). Cytoskeleton also participates in the regulation of host immune responses to infection by pathogens. Viruses interact with the cytoskeleton and motor proteins at different stages during their replication cycle (Słońska et al., 2021). The resistance mechanisms in the South American cassava were based on restriction in virus replication and virus movement (Sheat et al., 2019, 2021) which point in our case to cytoskeletal motor activity as a potential candidate gene for future studies. Additionally, two other gene ontologies, catalytic activity (GO:0003824) and structural molecule activity (GO:0005198), were previously observed to be upregulated in Namikonga, a common source of CBSD resistance, specifically at 2 and 4 days after inoculation (Amuge et al., 2017). Notably, our study did not identify common gene ontologies when compared with previous transcription studies on CBSD and whiteflies (Maruthi et al., 2014). This distinctive insight adds valuable knowledge to the intricate relationship between gene functions and viral interactions, shedding light on novel aspects of these complex biological processes.

## Conclusions

In summary, our study showed that CBSV titer had higher prevalence compared to UCBSV prevalence in the Cycle two population of genomic selection. CBSV was detected in 50.2% of clones, while UCBSV in 12.9%. Genotypic effects played a significant role, especially for UCBSV titer. GWAS studies revealed specific genomic regions linked to CBSV and UCBSV titer. Notably, 21 SNP markers located on chromosomes 10, 13,17 and 18 explained 43.14% of CBSV variation, while 25 markers located on chromosomes 1, 2, 4, 5, 8, 11, 12, 16 and 18 explained 70.71% of UCBSV variation. Gene ontology analysis highlighted diverse gene functions, with prominent roles in transporter and catalytic activities. The study provides valuable insights into virus prevalence, genetics, and molecular functions in cassava plants, aiding targeted breeding strategies. We recommend that breeding programs prioritize developing two independent strategies for CBSD: one targeting CBSV and the other UCBSV, given their association with distinct genomic regions. Additionally, genomic regions explaining larger proportions of phenotypic variance should be investigated further in independent populations, as they can be utilized in marker-assisted selection or genomic prediction.

## Data availability statement

The datasets presented in this study can be found in online repositories. The names of the repository/repositories and accession number(s) can be found below: https://cassavabase.org/breeders/trial/6707,https://cassavabase.org/breeders/trial/7071, https://cassavabase.org/breeders/trial/7795,https://cassavabase.org/breeders/trial/7746.

## Author contributions

LN: Conceptualization, Data curation, Formal Analysis, Investigation, Methodology, Validation, Visualization, Writing – original draft, Writing – review & editing. SS: Data curation, Investigation, Methodology, Supervision, Validation, Writing – original draft, Writing – review & editing. SW: Funding acquisition, Methodology, Project administration, Resources, Supervision, Writing – original draft, Writing – review & editing. AO: Data curation, Formal Analysis, Visualization, Writing – original draft, Writing – review & editing, Methodology. RK: Conceptualization, Funding acquisition, Investigation, Methodology, Project administration, Resources, Visualization, Writing – original draft, Writing – review & editing. J-LJ: Conceptualization, Funding acquisition, Investigation, Methodology, Project administration, Resources, Supervision, Validation, Writing – original draft, Writing – review & editing.

## Supplementary material

Supplementary Figure 1 Manhattan and Quantile-quantile (Q-Q) plots of genome-wide association studies for CBSDs3 **(A, B)**, CBSDs6 **(C, D)** and CBSDs12 **(E, F)** with CBSV titer as a covariate. CBSDs3 = cassava brown streak foliar severity at 3 MAP; CBSDs6 = cassava brown streak foliar severity at 3 MAP; CBSDs12= cassava brown streak root severity and CBSV titer = Cassava brown streak virus titer. Orange horizontal line indicates Bonferroni genome wide significance level [-log_10_(0.05/number of markers)].

Supplementary Figure 2 Manhattan and Quantile-quantile (Q-Q) plots of genome-wide association studies for CBSDs3 **(A, B)**, CBSDs6 **(C, D)** and CBSDs12 **(E, F)** with UCBSV titer as a covariate. CBSDs3 = cassava brown streak foliar severity at 3 MAP; CBSDs6 = cassava brown streak foliar severity at 3 MAP; CBSDs12= cassava brown streak root severity and UCBSV titer = Uganda cassava brown streak virus titer. Orange horizontal line indicates Bonferroni genome wide significance level [-log_10_(0.05/number of markers)].
