# Supplementary figures and images for: Genetic complexity of cassava brown streak disease: insights from qPCR-based viral titer analysis and genome-wide association studies
Source: Front Plant Sci. 2024 Mar 13;15:1365132. doi: 10.3389/fpls.2024.1365132 (PMC10965612; doi:10.3389/fpls.2024.1365132)

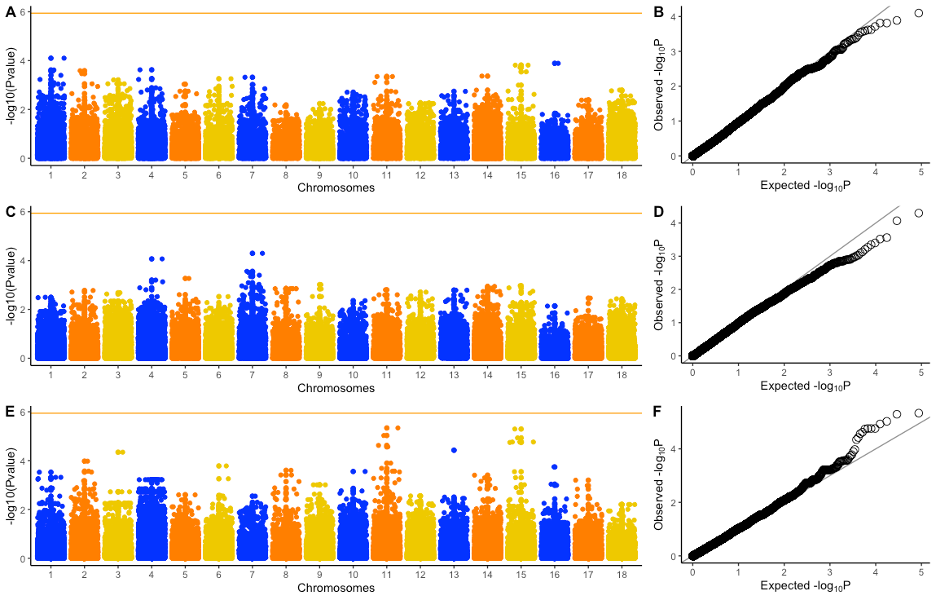

Supplement: Supplementary Figure 1 — Manhattan and Quantile-quantile (Q-Q) plots of genome-wide association studies for CBSDs3 (A, B), CBSDs6 (C, D) and CBSDs12 (E, F) with CBSV titer as a covariate. CBSDs3 = cassava brown streak foliar severity at 3 MAP; CBSDs6 = cassava brown streak foliar severity at 3 MAP; CBSDs12= cassava brown streak root severity and CBSV titer = Cassava brown streak virus titer. Orange horizontal line indicates Bonferroni genome wide significance level [-log10(0.05/number of markers)]. [file Image_1.jpeg]

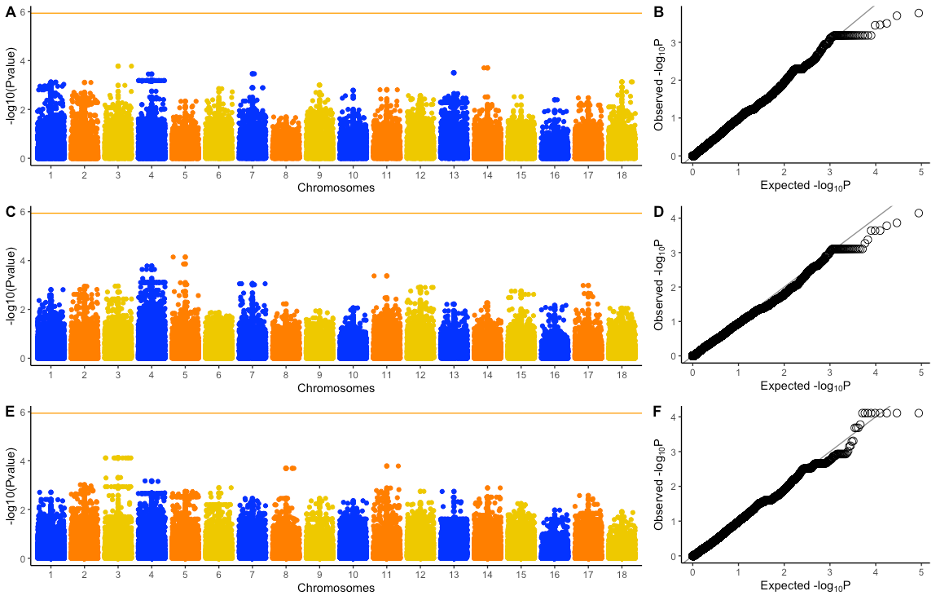

Supplement: Supplementary Figure 2 — Manhattan and Quantile-quantile (Q-Q) plots of genome-wide association studies for CBSDs3 (A, B), CBSDs6 (C, D) and CBSDs12 (E, F) with UCBSV titer as a covariate. CBSDs3 = cassava brown streak foliar severity at 3 MAP; CBSDs6 = cassava brown streak foliar severity at 3 MAP; CBSDs12= cassava brown streak root severity and UCBSV titer = Uganda cassava brown streak virus titer. Orange horizontal line indicates Bonferroni genome wide significance level [-log10(0.05/number of markers)]. [file Image_2.jpeg]
